# The Improvement Readiness scale of the SCORE survey: a metric to assess capacity for quality improvement in healthcare

**DOI:** 10.1186/s12913-018-3743-0

**Published:** 2018-12-17

**Authors:** Kathryn C. Adair, Krystina Quow, Allan Frankel, Paul J. Mosca, Jochen Profit, Allison Hadley, Michael Leonard, J. Bryan Sexton

**Affiliations:** 1 Duke Patient Safety Center, Duke University Health System, Durham, NC USA; 2 Department of Psychiatry, Duke University School of Medicine, Durham, NC USA; 3 Duke University School of Medicine, Duke University Health System, Durham, NC USA; 4 Safe and Reliable Healthcare, Evergreen, Colorado USA; 5 Duke Network Services, Duke University Health System, Durham, NC USA; 6 Department of Surgery, Duke University School of Medicine, Durham, NC USA; 7 Division of Neonatal Developmental Medicine, Department of Pediatrics, Stanford University, Stanford, CA USA; 8 Division of Pediatric Critical Care Medicine, Department of Pediatrics, Duke Children’s Hospital and Health Center, Durham, NC USA

**Keywords:** Improvement readiness, SCORE, quality improvement, Qualitative responses, Learning environment, Safety culture survey

## Abstract

**Background:**

Quality improvement efforts are inextricably linked to the readiness of healthcare workers to take them on. The current study aims to clarify the nature and measurement of Improvement Readiness (IR) by 1) examining the psychometric properties of a novel IR scale, 2) assessing relationships between IR and other safety culture domains 3) exploring whether IR differs by healthcare worker demographic factors, and 4) examining linguistic differences in word type use between high and low scoring IR work settings from their free text responses.

**Methods:**

Of 13,040 eligible healthcare workers across a large academic health system, 10,627 (response rate 81%) completed the 5-item IR scale, demographics, safety culture scales, and two open-ended questions. Psychometric analyses, correlations and ANOVAs tested the properties of IR. Linguistic Inquiry Word Count software assessed comments from open-ended questions.

**Results:**

The IR scale exhibited strong psychometric properties and a one factor model fit the data well (Cronbach’s alpha = .93; RMSEA = .07; CFI = 99; TLI = .99). IR scores differed significantly by role, shift, shift length, and years in specialty. IR correlated significantly and in expected directions with safety culture scales. Linguistic analyses revealed that people in low versus high IR work settings used significantly more words in their responses, and specifically more past tense verbs (e.g., “ignored”), negative emotion words (e.g., “upset”), and first person singular (“I”). Workers from high IR work settings used significantly more positive emotions words (e.g., “grateful”) and social words (e.g., “team”).

**Conclusion:**

The IR scale exhibits strong psychometric properties, is associated with better safety and teamwork climate, lower burnout, and predicts linguistic differences in high versus low IR groups.

## Background

Overwhelmed healthcare workers struggle to find time for meals and bathroom breaks, let alone to initiate or fully engage in quality improvement projects*.* Yet, the safety and reliability of healthcare delivery depends on healthcare workers having sufficient capacity and support to engage in continuous self-reflection and quality improvement. How do leaders know if groups in their organization are capable of useful self-reflection, and ready to engage in meaningful quality improvement? To date, no validated scale exists to specifically measure Improvement Readiness (IR), or the ability of a work-setting to effectively participate in continuous learning.

Continuous learning around quality improvement can take many forms, such as integrating lessons learned from other work settings, incorporating insights and ideas of workers into the delivery of care, and learning from defects. The acts of self-reflection and learning are essential ingredients in all quality improvement, from introducing a new labeling procedure to launching (and subsequently fine-tuning) a system-wide electronic medical record. Entities and work settings vary considerably with respect to how well equipped they are to support continuous learning. In order to facilitate IR, many healthcare systems build organization-wide infrastructures to enable and support integrated assessments and feedback. These include explicit support from leadership and co-workers and protected time for continuous learning either on-the-job or via lectures and conferences [1–3]. Ideally, there are processes in place at the work-setting level to maximally learn, whether from deficits or other opportunities as they arise [4]. Conversely, key barriers to learning include time and financial constraints, lack of support from leadership and peers, and a negative workplace culture with insufficient processes to facilitate employees’ learning [1, 2, 5].

When IR is high, workers feel there are processes and norms within a work setting that encourage employees to learn, improve, and sustain quality efforts. Embedded within the infrastructure of the workplace environment, employees are empowered to recognize and address improvement opportunities while celebrating improvement efforts and positive results. Furthermore, areas with a high degree of IR are environments of psychological safety in which employees feel their opinions and suggestions are heard, that there is the capacity for them to be acted upon, and that improvements do occur [6–8].

 Despite IR playing a vital role in healthcare quality, and even though the business literature lauds the importance of leaders who create IR, relatively little empirical attention has been paid to this construct in healthcare. Components of IR appear to somewhat overlap with constructs more often discussed in business, such as ‘organizational readiness for change’, ‘change readiness’, and ‘readiness for implementation’ [9–11]. A review of these constructs finds two overarching components: the motivational or attitudinal aspect of change (e.g., willingness) and the ability (e.g., being trained) [9]. The current conceptualization of IR was theoretically drawn from the ‘continuous learning and improvement’ component of Frankel and Leonard’s comprehensive approach to healthcare quality and safety [12]. It specifies that one’s ability to engage in continuous learning in one’s work setting is central, and even a *precondition for*, meaningful engagement in quality improvement. Although the IR construct overlaps with notions of ‘ability’ described in related work, scholars have not emphasized continuous learning and the conditions that support it. Despite a need for psychometrically robust scales for this construct, existing change or improvement-related measures suffer from limited evidence of reliability or validity [9]. The current study helps address this gap by making use of safety culture survey data collected across a large academic health system, which includes a novel measure of IR, five safety culture domains (e.g., teamwork and safety climate), and open-ended text box responses.

Safety culture surveys often end with open-ended text boxes for optional comments, however, no study, to our knowledge, has used the text from these types of comments in an empirical article to examine linguistic differences across various groups in healthcare. Linguistic research has found that underlying psychological factors appear to express themselves through word choice [13–16]. Based on prior research, we expected that healthcare workers who work in settings with low IR are likely to reveal their lack of resources and higher stress, through a less healthy pattern of language. Therefore, we expected workers from low IR work settings to more frequently use first person singular pronouns [13, 17, 18], words that focus on the past (i.e., past verb tense, indicative of being in reactive mode) [19–21], and negative emotions words [14], and to less frequently use first person plural pronouns (i.e., “we/our/us”) and positive emotions [14, 22], and social words (e.g., “team”) [23] compared to high IR work settings. Finally, since open-ended text prompts may be one of the few opportunities for staff members from under-resourced or stressed work setting to speak up about concerning issues, we expected workers from low IR work settings to submit longer responses (i.e., more words).

The current study had four aims which each served to clarify the nature and measurement of IR. The first aim was to examine the psychometric properties of a novel IR scale. The second aim was to assess relationships between IR and other safety culture measures from *SCORE* [7]. We expected IR to correlate positively with *Local Leadership, Safety Climate, Teamwork Climate,* and to correlate negatively with *Personal Burnout, Burnout Climate, and problems with Work-life Climate* [7]*.* The third aim was to explore whether IR differs by healthcare worker role, shift, shift length, and years in specialty. The fourth aim was to examine linguistic differences in word type use between high and low scoring IR work settings in their free text responses.

## Methods

### Design and study population

This is a cross sectional study of 2016 survey data sent to 13,040 healthcare workers across 440 work settings within one academic health system as part of the Safety, Communication, Operational, Reliability, and Engagement (SCORE) survey [7]. This study was approved by the Duke University Health System Institutional Review Board.

All staff with 50% or greater full-time equivalent commitment to a specific work-setting for at least four consecutive weeks were asked to participate. Work settings with five or more respondents and a response rate of at least 40% were included in the aggregated analyses (i.e., domain level correlations), resulting in a sample of 396 work settings (90%).

### Measurement of improvement readiness (IR)

**Fig. 1:**
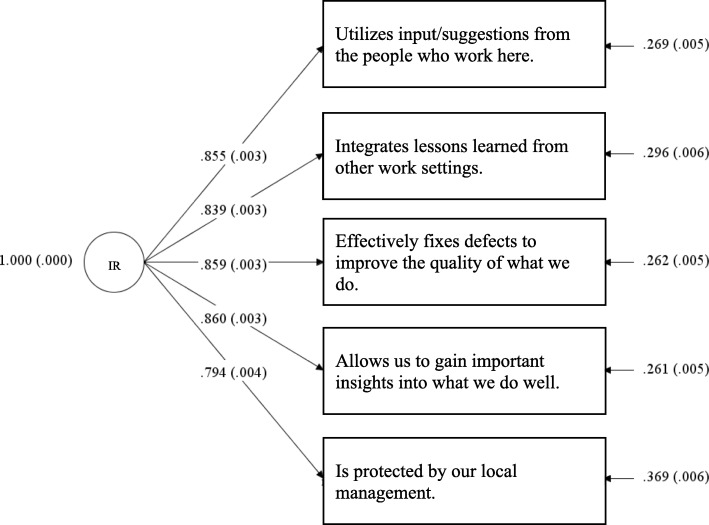
Standardized Factor Loadings for Improvement Readiness Scale

Improvement Readiness measure is a sub-scale within the SCORE survey, which can be used on its own or as a part of SCORE [7]. The scale’s five items were presented to the respondent as phrases that follow an initial prompt that says: “The learning environment in this work setting …”, e.g., “Integrates lessons learned from other work settings.” Respondents are asked to rate the items on a 1–5 scale, (1 = disagree strongly, 5 = agree strongly). See Fig. 1.

### SCORE survey

Scales assessing Work-life Climate, Teamwork Climate, Safety Culture, Burnout Climate, Personal Burnout, and Local Leadership, were also measured within the SCORE survey [7].

### Statistical analysis

Improvement Readiness scale scores were calculated by taking the mean of the five items for each respondent using the 5-point scale. To assess the extent respondents perceived IR favorably across their work settings, or in other words the IR climate, an aggregate percentage of positive responses was computed by calculating the percentage of respondents in each work setting who scored greater than or equal to a 4 (e.g., those who, on average, “agreed slightly” or “agreed strongly”). IR aggregated climate scores were only used in the current study to examine associations with other safety culture scores, which were also computed using the same technique.

Confirmatory factor analysis (CFA) with maximum likelihood estimation was used to examine the fit of a one-factor model for the 5 IR item scale. Fit was assessed using the following indices: Root Mean Square Error Approximation (RMSEA; < .08 considered adequate fit) [24], Confirmatory Fit Index (CFI), and Tucker-Lewis Fit Index (TLI; CFI and TLI’s > .95 considered acceptable fit) [25]. Internal reliability was assessed using Cronbach’s alpha (α; > .70 is considered acceptable) [26]. Analysis of variance (ANOVA) was used to test for differences on the IR scale by healthcare worker role, shift, shift length, and years in specialty. A Random Effects ANOVA tested both within work-setting variance and between work setting variance for the IR scale. An Intraclass Correlation Coefficient (ICC) was computed to assess the extent of dependence or clustering of IR scores based on work-setting, which supports the use of aggregating scores at the work setting level [27]. Statistical analyses were performed using IBM SPSS V.24 [28] and Mplus version 7.4 [29].

### Linguistic inquiry and word count analysis of comments

Linguistic word count patterns were assessed with the Linguistic Inquiry and Word Count (LIWC) software program, version 2015 [30]. LIWC uses an internal dictionary of 6400 words and word stems to process text files to calculate the percentages of total words that fit into 80 language categories. The categories include standard linguistic dimensions, social and psychological processes, affect-related words, and non-psychological processes [31]. The word counts are expressed as a percentage of the total number of words used thus controlling for the length of the input text file.

The current study hypothesized different calculated word percentages in specific pre-determined linguistic categories for open-ended comments from work settings coming from the highest versus lowest 10 % across the current sample in Improvement Readiness. Top and bottom deciles were selected to facilitate thematic analysis of these comments for a separate project, ensuring that every comment subjected to LIWC in the current study was also read by multiple people. The SCORE survey included two open ended questions: “Please share something you have seen make a positive impact on the culture in your work area that you recommend continue”, and “Do you have any other comments, questions, or concerns?”. To maximize the amount of text examined for each hypothesis, the responses to both questions were combined for each respondent.

To visualize the frequency of words used, Wordles were created for the high and low IR work settings. Wordles are word clouds that display the size of a word proportionally to the number of times the word appears in a section of text.

T-tests were used to compare linguistic category count variables for the high and low IR work settings. For T-tests that failed Levene’s test for equality of variances, we report values that did not assume equal variances. 

## Results

### Respondent demographics

Electronic surveys were returned by 10,627 out of 13,040 possible survey respondents (overall response rate 81%). Table 1 presents demographic data for respondents.

**Table 1:** Respondent Demographics and Improvement Readiness Cronbach’s α

	N	Cronbach’s α	% of Total
Role			
Nurse	3367	0.919	31.7%
Physician: Attending	1036	0.926	9.7%
Technologist (e.g., Surg., Lab, Rad.)	869	0.933	8.2%
Other	689	0.941	6.5%
Technician (e.g., PCT, Surg., Lab, EKG, Rad.)	567	0.937	5.3%
Administrative Support (Administrative Asst., Work setting Coordinator, etc.)	542	0.935	5.1%
Advance Practice Provider (PA/NP/CRNA/Nurse Clinician)	503	0.922	4.7%
Clinical Support (Medical Assistant, CMA, EMT, etc.)	500	0.922	4.7%
Nurse’s Aide	489	0.930	4.6%
Therapist (RT, PT, OT, SLP)	462	0.910	4.3%
Administrator/Manager/Supervisor	388	0.872	3.7%
Physician: Resident	275	0.912	2.6%
Pharmacist	198	0.927	1.9%
Physician: Fellow	157	0.954	1.5%
Clinical Social Worker/Case Manager	130	0.943	1.2%
Dietician/Nutritionist	51	0.850	0.5%
Environmental Services	41	0.964	0.4%
Psychologist	20	0.800	0.2%
Missing	343	0.925	3.2%
Years In Specialty			
Less than 6 months	1264	0.918	11.9%
6 to 11 months	2184	0.928	20.6%
1 to 2 years	1974	0.934	18.6%
3 to 4 years	1410	0.921	13.3%
5 to 10 years	2423	0.929	22.8%
11 to 20 years	877	0.910	8.3%
21 years or more	404	0.901	3.8%
Missing	91	0.942	0.9%
Shift			
Day	7235	0.928	68.1%
Night	1269	0.925	11.9%
Other	946	0.933	8.9%
Swing	1000	0.908	9.4%
Missing	177	0.923	1.7%
Shift Length			
10 h	1402	0.929	13.2%
12 h	3482	0.916	32.8%
8 h	4320	0.932	40.7%
Flex	321	0.927	3%
Other	941	0.929	8.9%
Missing	161	0.924	1.5%
Total	10,627	0.930	100%

Values on the arrows from IR to items represent standardized factor loadings and their standard errors, in parentheses. Values on the far right side of the figure represent residual variances for the items and their standard errors, in parentheses. The scale’s five items were presented as phrases that follow an initial prompt: “The learning environment in this work setting…”

The top three respondent groups were registered nurses (31.7%; *n* = 3367), attending physicians (9.7%; *n* = 1036), and technologists (8.2%; *n* = 869). A subset of respondents (3.2%) did not identify with one of the listed healthcare worker roles. Respondents were predominantly day-shift workers (68.1%), with diversity in years of experience in their specialty and shift length. Missing data for each of the items ranged from 0.9 to 3.2%.

### Aim 1) psychometrics and confirmatory factor analysis

**Fig. 2:**
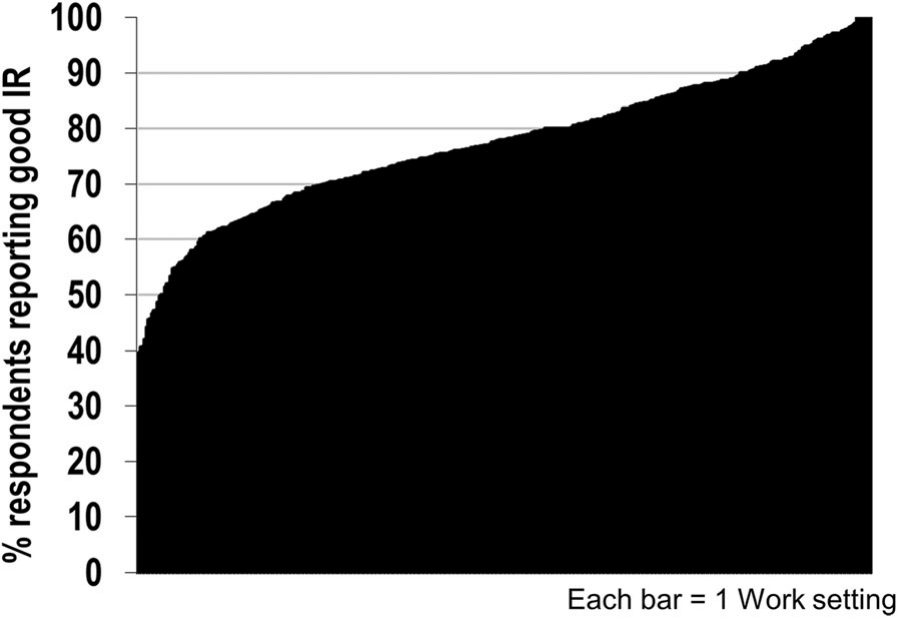
Percent of Respondents Reporting Good IR across 396 Work Settings

The mean IR score across those who completed the scale (*n* = 10,154) was 4.08 (SD = .97). The mean percent positive IR climate score across the 396 work settings was 77.28 (SD = 12.74) and work settings ranged from 38.91 to 100% positive IR climate (See Fig. 2). The overall sample’s Cronbach’s alpha, an index of internal reliability, was .93. Above .70 is considered acceptable, particularly for shorter scales [26]. The alpha ranged from .80 to .96 across various demographic groups (see Table 1).

A one factor model of the scale produced good fit to the observed data (RMSEA = .07, 90% CI: .06–.078; CFI = .99; TLI = .99). Standardized factor loadings ranged from .79 to.86 (See Fig. 1).

A Random Effects ANOVA of the entire sample revealed significant variance within (.847) and between (.093) work-setting variance in IR scores, *p*-values < .001. The computed ICC was 0.10, indicating 10% of the variance in IR scores can be attributed to between work-setting characteristics. In other words, there was significant clustering of IR responses within work settings, reflecting a non-trivial level of consensus at the work setting level.

### Aim 2) associations with safety culture and burnout scales

IR significantly predicted all other Safety Culture scales in expected directions. Specifically, IR was positively correlated with Local Leadership, Safety Climate, and Teamwork Climate. IR was negatively correlated with Personal Burnout, Burnout Climate and problems with Work-life Climate (See Table 2). Correlation coefficients ranged from .756 (Safety Climate) to .405 (Work-life Climate).

**Table 2:** Correlation matrix for Improvement Readiness and additional healthcare climates surveyed. Cronbach’s alpha for each domain included in the diagonal

Variable	1	2	3	4	5	6	7
1. Improvement Readiness	**(.93)**						
2. Work-life Climate	.405*	**(.83)**					
3. Teamwork Climate	.661*	.367*	**(.76)**				
4. Safety Climate	.756*	.424*	.733*	**(.87)**			
5. Burnout Climate	−.642*	−.527*	−.661*	−.695*	**(.90)**		
6. Personal Burnout	−.690*	−.545*	−.636*	−.656*	.813*	**(.92)**	
7. Local Leadership	.727*	.367*	.607*	.706*	−.527*	−.567*	**(.94)**

*p <  0.01 level (2-tailed)

All scores were aggregated at the work-setting level

### Aim 3) IR by role, shift, shift length, and years in specialty

Univariate ANOVA demonstrated significant differences in the IR scale between healthcare worker role (F(18, 10,135) = 12.92, *p* <  0.001), shift (F(4, 10,154) = 11.94, *p* <  0.001), shift length (F(5, 10,155) = 10.27, *p* <  0.001), and years in specialty (F(7, 10,155) = 13.54, *p* <  0.001).

Scheffé post hoc tests revealed Administrators/Managers/Supervisors reported significantly better IR than 13 of the 18 possible roles. Administrators/Managers/Supervisors reported statistically equivalent IR to Clinical Support Workers (e.g., Medical Assistant, EMT), Dietician/Nutritionists, Physician Fellows, and Environmental Services. Psychologists and Clinical Social Workers/Case Managers reported significantly lower IR than all other possible roles in the current sample.

Scheffé post hoc tests revealed day shift workers reported higher IR than night and ‘other’ shift workers. Swing workers reported higher IR than ‘other’ shift workers. Eight-hour shift workers reported higher IR than 10-, and 12-h shift workers, and those who described their shift as ‘other’.

Workers with fewer years in specialty generally report higher IR. Specifically, those less than 6 months in their specialty reported significantly higher IR than every other length in specialty, except for those 6–11 months in specialty.

### Aim 4) linguistic analyses

Percent positive IR scores (percent that on average, agreed with IR items) were computed for each work-setting. The top and bottom 10% of work settings were identified. The work settings in the lowest 10% (39 work settings) had a mean IR score of 52.51 (SD = 6.54). The work settings in the highest 10% (39 work settings) had a mean IR score of 97.15 (SD = 1.92).

**Fig. 3:**
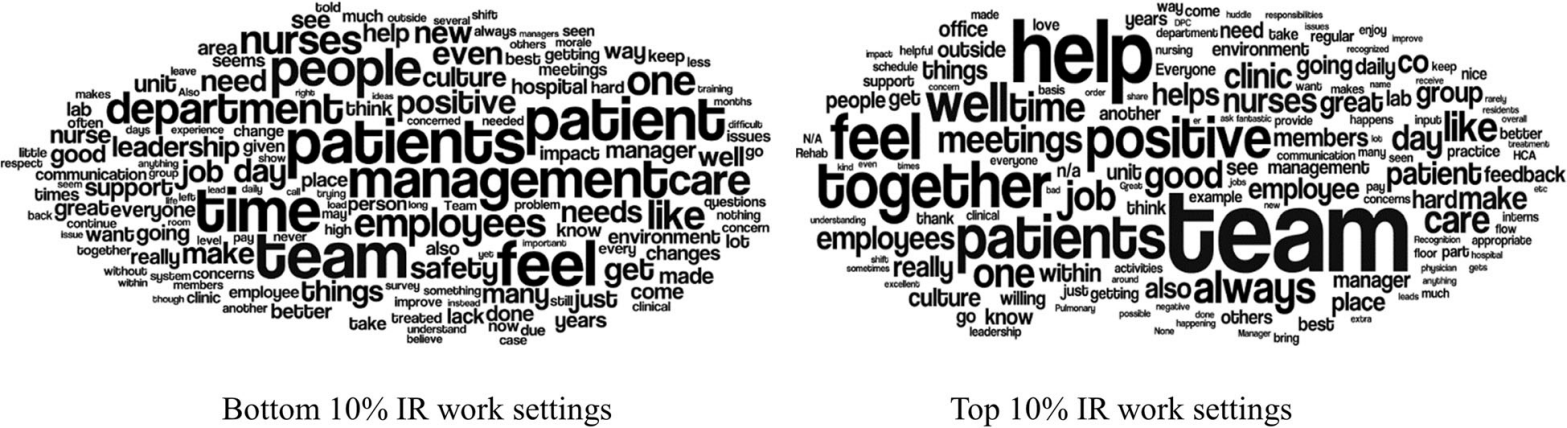
Wordles of Comments from Low (left) and High (right) IR Work Settings

Three hundred and fifty-five workers provided comments from the low IR work settings (31.4% of the 1129 who completed the survey from these groups). One hundred and forty-nine workers provided comments from high IR work settings (27.3% of the 536 who completed the survey from these groups). Wordles, which graphically depict word frequency, were created for the high and low IR groups, and can be found in Fig. 3. Representative comments from high and low IR work settings can be found in Table 3.

**Table 3:** Representative Comments from the Top and Bottom 10% IR groups

Question 1: Please share something you have seen make a positive impact on the culture in your work area that you recommend continue.	
Top 10% IR	
Our nurse manager talked with all of us about how to handle a conflict with another employee. She always leads by example and practices what she preaches. As a result, we have been able to come to each other when we are having a conflict. That allows opportunities for us to handle it on our own. It seems to bring us all closer together.	
Encouraging the nurses to raise any questions/concerns they have. It averts errors in ordering, and also provides teaching opportunities when the order is what we intended.	
Bottom 10% IR	
We are so burned out that if anything positive happens we have not had time to notice it.	
CAN NOT THINK OF ANY POSITIVE CHANGES. HOWEVER, SEVERAL NEGATIVE COME TO MIND!	
Question 2: Do you have any other comments, questions, or concerns?	
Top 10% IR	
This is a great place to work. The clinical staff are really a team. Everyone helps each other and they each create a positive environment.	
I am new here and felt very welcome and part of the team since day one. I feel very honored to work for this wonderful group. It feels like a group of friends who happen to work together more than co-worker relationships.	
Bottom 10% IR	
Feedback is rarely given to residents (attendings have other things to worry about) so the few instances when feedback is given have a disproportionate impact. We get feedback twice per year from the Program Director, and almost never outside of that setting (you almost never hear from attendings how you’re doing with/for their patients, in cases, in clinic, etc). The overall culture is to keep your head down and not rock the boat. This isn’t exactly conducive to raising issues when they come up. Despite generally difficult/stand-offish attending-resident relations, resident morale is ok because we all get along and work hard for each other.	
This place wants “yes people”. I feel upper management makes decisions and then only wants people who will implement the plans they already have made. My opinion counts for nothing. You are just the worker that helps them accomplish their goals.	

**Fig. 4:**
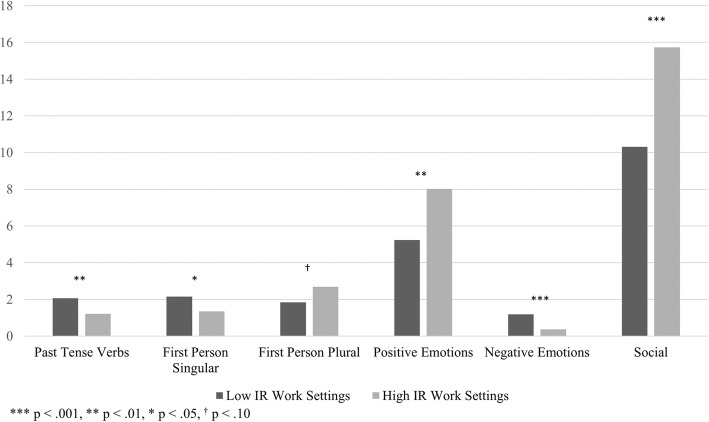
Word Counts for High and Low IR Work Settings

Table 4 and Fig. 4 show the linguistic analyses results. As hypothesized, workers from low IR work settings used significantly more words overall in their responses, as well as more past tense verbs, first person singular, and negative emotions words, compared to those from high IR work settings. As a follow-up to the negative emotion words result, exploratory analysis found the rates of the three negative emotion sub categories, anxiety, anger, and sadness, were significantly greater for low IR work settings than for high IR work settings.

**Table 4:** T-test results for LIWC analyses comparing high and low IR work settings

	*Low 10% IR Work settings*	*High 10% IR Work settings*	T	*p*-value
	Mean (SD)	Mean (SD)	df	
Word Count	80.99 (126.12)	29.01 (37.59)	7.06	< 0.001***
			469.41	
Past Tense Verbs	2.06 (3.45)	1.21 (2.70)	2.67	0.008**
			503	
First Person Singular	2.15 (4.19)	1.34 (2.95)	2.47	0.014*
			388.73	
First Person Plural	1.83 (5.48)	2.69 (4.44)	−1.85	0.065^†^
			339.35	
Positive Emotion	5.24 (9.10)	8.02 (10.35)	−2.85	0.005**
			248.39	
Negative Emotions	1.18 (2.10)	0.37 (1.18)	5.50	< 0.001***
			462.21	
Anxiety	0.24 (0.79)	0.10 (0.57)	2.28	0.023*
			377.27	
Anger	0.24 (1.02)	0.03 (0.21)	3.59	< 0.001***
			419.13	
Sadness	0.25 (0.83)	0.08 (0.55)	2.73	0.007**
			408.03	
Social	10.31 (10.74)	15.73 (13.83)	−4.28	<0.001***
			226.23	

*** *p* < .001, * *p* < .010, * *p* < .05, ^†^ *p* < .10

All word categories (except “Past Tense Verbs”) failed Levene’s tests for equality of variances; in these cases, equal variances were not assumed for calculating T and *p*-values

As hypothesized, workers from high IR work-settings used significantly more positive emotions words and social words. Wordles appear to reflect the findings from the linguistic analysis. For example, in Fig. 3, note the large size of the words, “together,” “team,” and “help” reflect that high IR work settings use more social words.

## Discussion

In this study we provide evidence for the psychometric soundness, reliability, and convergent and construct validity of a novel IR scale.

### Aim 1) examining the psychometric properties of a novel IR scale

The IR scale meets and exceeds established psychometric thresholds for reliability [26], and the single factor model provided good fit to the data, demonstrating construct validity [24, 25, 32]. Moreover, the IR construct appears to behave like a group norm or climate, as evidenced by the non-trivial clustering within a work setting.

### Aim 2) assessing relationships between IR and other safety culture scales

The IR scale was clearly associated with established safety culture and burnout scales in expected directions. Specifically, work settings with higher IR also report better *Local Leadership, Safety Climate, Teamwork Climate,* and *Work-life Climate*, as well as lower *Personal Burnout* and *Burnout Climate*. Work settings that are struggling with issues related to leadership, patient safety and teamwork are not yet at a place where they can meaningfully engage in learning to improve quality. Rather, and understandably, their efforts are focused on reacting to current deficits. The IR scale offers a window to managers and leaders as to which groups are best prepared to trial a new initiative or process to improve quality.

Of particular note is that the highest loading IR item was “The learning environment in this work setting allows us to gain important insights into what we do well.” This suggests that pausing and reflecting on what is going well is a key component of IR, which is interesting considering that improvement efforts often focus exclusively on what is *not* going well. The importance of positive reflection to gain insight was captured in the positive emotions linguistic finding, as well as in several comments from the top 10% IR group, e.g., “[My leader] does a fantastic job at checking in on employees to see what is going well, what can be improved, any concerns that need to be addressed. She follows up on each of these things. She writes a card to those who are recommended for recognition and hand delivers them! She is the best Manager I have ever had and feel fortunate to be under her. She is a rock in this department!!!!”.

### Aim 3) exploring whether IR differs by healthcare worker demographic factors

Roles, shift type, shift length, and years in specialty differentially predicted rates of IR. In general, those with fewer years in specialty, who work day shifts, and are 8-h shift workers, reported higher IR. If poor IR is to a group as poor work-life balance is to individuals, then it might represent available bandwidth to learn and participate in quality improvement. To that end, it isn’t surprising that many of the differences in IR by demographics look similar to their counterpart differences in poor work-life balance [33]. Future research would benefit from understanding what factors play a role for those reporting higher IR, so that these factors could be adopted more broadly. For example, it may be that those much earlier in their careers receive more support for their learning and development, that they receive more feedback on their progress, or that they are simply less burned out than those farther along.

Larger work settings generally have lower IR scores than smaller work settings. Specifically, the number of people in the lowest IR decile of work settings was twice as many as the number of people in the highest decile. Perhaps it is more difficult to coordinate improvements in larger groups. Larger groups are also more likely to have higher worker to management ratios and higher acuity patients, and therefore are less likely to have effective feedback and quality improvement discussions, which are components of IR.

### Aim 4) examining linguistic differences in word type use between high and low scoring IR work settings in their free text responses

Linguistically, we learned higher performing IR work settings speak less in the first person singular (I/me/my), and more in the first-person plural (we/our/us; at the level of a trend). This mirrors the established finding in linguistics research that greater first person singular use is a marker of depression, since emotional pain tends to draw attention to people’s own experiences [13, 14, 27]. In low IR work settings, this may reflect lacking control over one’s environment, leading to feelings of, anger, sadness, and anxiety, which were all word categories that were higher in low IR settings. High IR work settings used less past tense, indicating they are less likely to be in reactive mode, addressing prior deficits [19, 20]. As anticipated, high IR settings also used more social words (a marker of wellbeing [33]), and reinforced by the 44% of the variance shared between IR and Teamwork Climate. Finally, it is particularly noteworthy that high IR work settings used more positive emotion words, given that psychological research consistently shows that better mental health and wellness is associated with more positive emotion word use [34–36].

Significant differences in word use in the top and bottom IR deciles suggest, in combination with the ICC results and the strong correlations with personal burnout, that there is a *climate* of IR. In other words, IR appears to operate as a group norm, that reflects more well-being and ability to take on new initiatives. The ICCs suggest this through the clustering of IR scores within work settings, the burnout correlations suggest this is related to wellness, and the LIWC results provide additional linguistic support.

This study is limited in its use of self-report data which are at risk for response, selection, and social desirability biases. This was a study of one large academic health system, thus, findings may not generalize broadly to other healthcare systems, or outside of healthcare. The linguistic findings from this study are limited by using only the top and bottom deciles, which were selected to facilitate thematic analysis of these comments for a separate project, ensuring that every comment subjected to LIWC in the current study was also read by multiple people. We therefore excluded 80% of the potential comments from these analyses. Nevertheless, 504 individuals’ comments, which totaled 33,076 words, were analyzed in the current work. Although there were 80 LIWC categories that could have been analyzed, we limited our selection to the most theoretically relevant categories. We merged responses to two distinct open-ended question prompts in order to maximize the number of words available for each respondent. However, we did look at the comments for each prompt separately and found a similar pattern of results. Finally, we did not assess whether work settings higher on IR actually achieve greater quality improvement success, an area for future research.

## Conclusions

Successful quality improvement initiatives hinge on the readiness of work settings to take them on. People interested in assessing IR have strong support from this study for the use of the brief, interpretable and reliable IR scale. The IR scale exhibits robust psychometric properties, is associated with established safety culture scales in expected directions (i.e., better safety and teamwork climate, lower burnout), and predicts linguistic differences in high versus low IR groups. As part of a safety culture assessment, IR provides a novel insight into the ability of a group to improve, which is distinct from, e.g., teamwork norms or patient safety deficits. The IR scale can help determine, among work settings with otherwise similar safety culture profiles, where we can expect readiness for improvement and where we cannot.
